# Side-specific factors for intraoperative hemodynamic instability in laparoscopic adrenalectomy for pheochromocytoma: a comparative study

**DOI:** 10.1007/s00464-024-10974-w

**Published:** 2024-07-01

**Authors:** Tamer A. A. M. Habeeb, Marta Araujo-Castro, Massimo Chiaretti, Mauro Podda, Alberto Aiolfi, Igor A. Kryvoruchko, Mallikarjuna N. Manangi, Vishal Shelat, Abd-Elfattah Kalmoush, Mohamed Fathy Labib, Mohammed Hassan Elshafey, Sameh Mohamed Mahmoud Ibrahim, Mohamed Ibrahim Abo Alsaad, Hamdi Elbelkasi, Mohamed Ibrahim Mansour, Tamer Mohamed Elshahidy, Ibrahim A. Heggy, Rasha S. Elsayed, Alaa A. Fiad, Ahmed M. Yehia, Mahmoud Abdou Yassin, Mahmoud R. Elballat, Mohamed H. Hebeishy, Ahmed Khaled AboZeid, Mohamed Adel Ahmed Saleh, Abd Elwahab M. Hamed, Amr A. Abdelghani, Bassam Mousa

**Affiliations:** 1 Department of General Surgery, Faculty of Medicine, Zagazig University, Zagazig, Egypt; 2 Neuroendocrinology & Adrenal Unit of the Endocrinology & Nutrition Department, Hospital Universitario Ramón y Cajal, Madrid, Spain; 3 Ramón y Cajal Research Institute (IRYCIS), Madrid, Spain; 4 Department of General Surgery Specialties and Organ Transplant, Faculty of Pharmacy and Medicine, Sapienza Rome University, Rome, Italy; 5 Department of Surgical Science, University of Cagliari, Cagliari, Italy; 6 Department of Biomedical Sciences for Health, Milan, Italy; 7 Surgery Department #2, Kharkiv National Medical University, Kharkiv, Ukraine; 8 Department of General Surgery, Bangalore Medical College and Research Institute, Bengaluru, India; 9 General Surgery, Tan Tock Seng Hospital, Singapore, Singapore; 10 General Surgery Department, Faculty of Medicine, Al-Azher University, Cairo, Egypt; 11 Lieutenant Colonel Doctor, Armed Forces College of Medicine, Cairo, Egypt; 12 General Surgery Department-Faculty of Medicine, Merit University, Sohag, Egypt; 13 Mataryia Teaching Hospital, GOTHI, Cairo, Egypt

**Keywords:** Hemodynamic instability, Laparoscopic adrenalectomy, Pheochromocytoma, Transperitoneal, Adrenalectomy

## Abstract

**Background:**

Adrenalectomy for pheochromocytoma (PHEO) is challenging because of the high risk of intraoperative hemodynamic instability (HDI). This study aimed to compare the incidence and risk factors of intraoperative HDI between laparoscopic left adrenalectomy (LLA) and laparoscopic right adrenalectomy (LRA).

**Methods:**

We retrospectively analyzed two hundred and seventy-one patients aged > 18 years with unilateral benign PHEO of any size who underwent transperitoneal laparoscopic adrenalectomy at our hospitals between September 2016 and September 2023. Patients were divided into LRA (*N* = 122) and LLA (*N* = 149) groups. Univariate and multivariate logistic regression analyses were used to predict intraoperative HDI. In multivariate analysis for the prediction of HDI, right-sided PHEO, PHEO size, preoperative comorbidities, and preoperative systolic blood pressure were included.

**Results:**

Intraoperative HDI was significantly higher in the LRA group than in the LLA (27% vs. 9.4%, *p* < 0.001). In the multivariate regression analysis, right-sided tumours showed a higher risk of intraoperative HDI (odds ratio [OR] 5.625, 95% confidence interval [CI], 1.147–27.577, *p* = 0.033). The tumor size (OR 11.019, 95% CI 3.996–30.38, *p* < 0.001), presence of preoperative comorbidities [diabetes mellitus, hypertension, and coronary heart disease] (OR 7.918, 95% CI 1.323–47.412, *p* = 0.023), and preoperative systolic blood pressure (OR 1.265, 95% CI 1.07–1.495, *p* = 0.006) were associated with a higher risk of HDI in both LRA and LLA, with no superiority of one side over the other.

**Conclusion:**

LRA was associated with a significantly higher intraoperative HDI than LLA. Right-sided PHEO was a risk factor for intraoperative HDI.

**Supplementary Information:**

The online version contains supplementary material available at 10.1007/s00464-024-10974-w.

Pheochromocytoma (PHEO) is an adrenomedullary chromaffin cell tumour that releases catecholamines [1]. The general population incidence of PHEO is 0.05–0.1%; however, it is higher in the hypertensive population, with a clinical presentation ranging from asymptomatic to sudden death [2–5]. Emerging technologies have evolved to include minimally invasive laparoscopic adrenalectomy, leading to an excellent view, fine dissection, and less manipulation, with lower morbidity and mortality [6]. The laparoscopic adrenalectomy technique comprises various laparoscopic transabdominal, laparoscopic retroperitoneal, and robotic procedures [7–9]. Gagner et al. [10] were the first to discuss transperitoneal laparoscopic adrenalectomies. Since then, many surgeons have recommended transperitoneal laparoscopic adrenalectomy owing to its familiar anatomy and wide working space [11].

Pheochromocytoma surgery remains challenging for surgeons and anesthesiologists because it is associated with hemodynamic instability (HDI) risk, presenting with sudden intraoperative hypertension, intraoperative hypotension, or prolonged hypotension after pheochromocytoma removal [2, 12–14]. Intraoperative HDI varies between studies and may reach up to 43.8% due to variations in preoperative medical preparation, anaesthesia protocol, and variable anatomical position of both adrenals [12, 15, 16]. The right adrenal gland has partial retrocaval localization near the liver and duodenum and drains by the short right adrenal vein to the inferior vena cava posterolaterally, which can easily bleed and is challenging to control during laparoscopic right adrenalectomy (LRA). All these factors make the LRA technically more complex, with a higher intraoperative HDI risk [17, 18]. On the other side, the left adrenal gland has a longer left adrenal vein close to the left renal hilum, pancreatic tail, spleen blood vessels, and the spleen’s inflexible character. Consequently, these factors make laparoscopic left adrenalectomy (LLA) complex, and a study showed that perioperative HDI occurs exclusively in the LLA [19].

Numerous HDI risk factors have been reported in the literature during laparoscopic adrenalectomy for pheochromocytoma, but the results were inconclusive or did not specifically address the side-specific factors for intraoperative HDI [2, 15, 20–25]. This diversity might be due to unstandardized anesthesiological and surgical management and different definitions of HDI. Preoperative evaluation of these risk factors is of great value for clinicians in evaluating the risk of HDI, developing a surgical plan, reducing the HDI rate, and improving the intraoperative situation and postoperative outcomes [26].

Variations in patient outcomes during LRA or LLA for managing pheochromocytoma resection have not been evaluated for the incidence and risk factors of intraoperative HDI differences between the two approaches in a large cohort of patients. Considering this background, our study aimed to fill this gap by comparing the incidence and risk factors of intraoperative HDI between LLA and LRA in a large cohort of patients with unilateral benign pheochromocytomas.

## Patients and methods

The study protocol was approved by the University Research Ethics Board (IRB Number: 11160) and was registered at www.clinicaltrials.gov (NCT06064370). The study followed the relevant STROCSS Guidelines: Strengthening the reporting of cohort studies on surgery [27].

### *The study team did not plan ‘*a priori*’ protocol for this study*

#### Study design and eligibility criteria

**Fig. 1:**
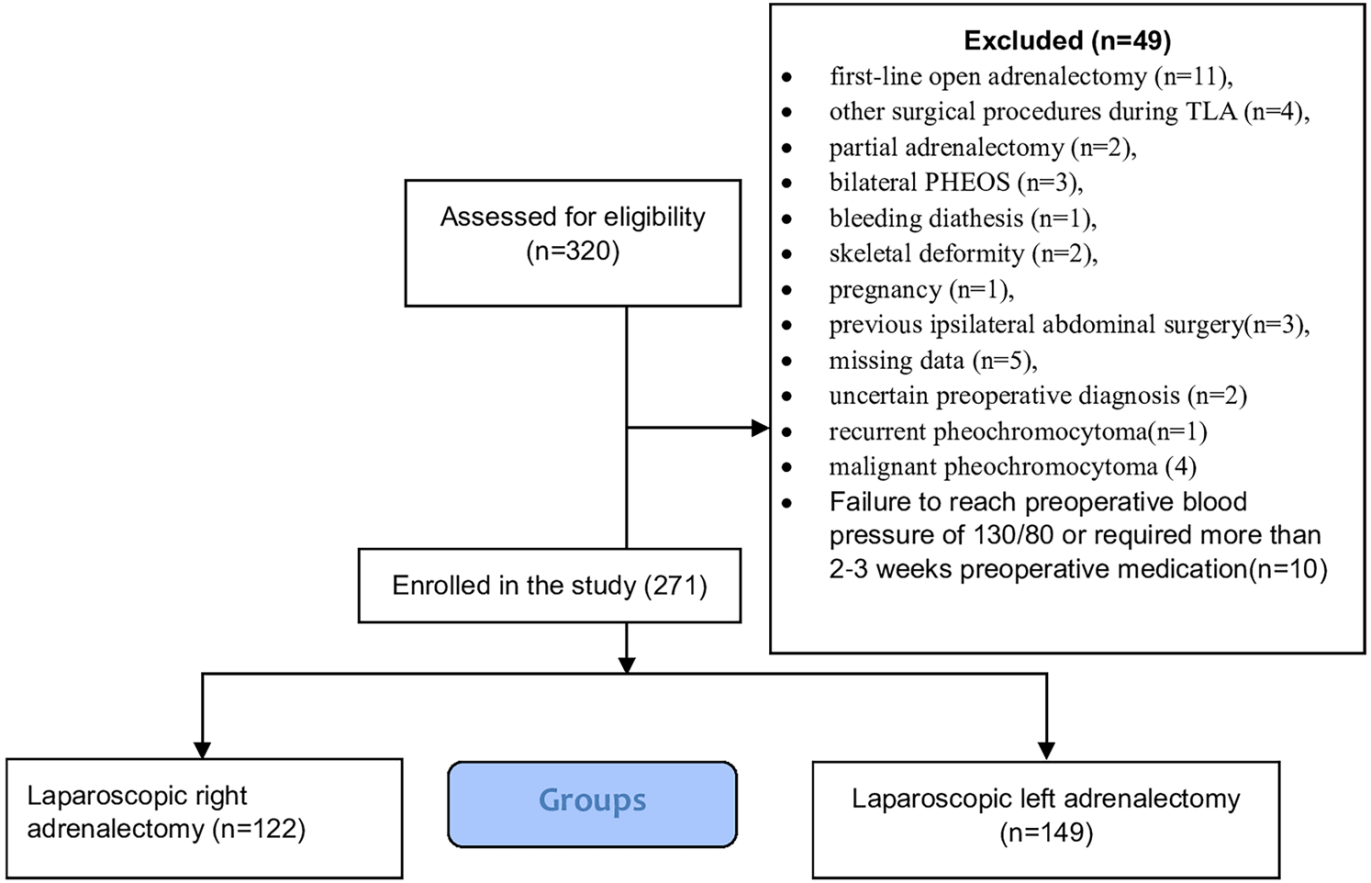
Flow diagram of inclusion and exclusion criteria of studied patients

A retrospective analysis of Consecutive patients with unilateral benign pheochromocytoma of any size > 18 years who underwent transperitoneal laparoscopic adrenalectomy at our hospitals between September 2016 and September 2023. The diagnosis was confirmed biochemically (plasma or urinary catecholamine and fractionated metanephrines), radiologically [computed tomography (CT)], and magnetic resonance imaging (MRI) [28]. Figure 1 shows a flow chart of the study patients’ inclusion and exclusion criteria.

### Variables collection

One of the participating surgeons collected the variables from the surgical and anaesthesia charts. The preoperative data of the patients, including demographic characteristics (age, sex, smoking, and body mass index), comorbidities [Diabetes Mellitus(DM), hypertension, coronary heart disease (CHD)], tumour characteristics (tumour side, size, and retrocaval position), preoperative symptoms, Preinduction hemodynamic data (systolic and diastolic blood pressure), Preoperative medical preparation (alpha-blockers including Bunazocin, Doxazocin, Phenoxybenzamine and Beta-blocker), number of antihypertensive drugs, 24 h urine analysis of epinephrine (*N* ≤ 24 mg/24 h), Nor epinephrine (*n* ≤ 66 mg/24 h), fractionated metanephrine, and normetanephrine (*n* ≤ 1.6 ng/ml). Intraoperative variables included operative time (min), estimated blood loss (ml), blood transfusion, intraoperative complications, intraoperative Hemodynamic Instability (incidence, form, number of episodes, and systolic blood pressure during hypertensive episodes) and conversion (incidence and causes). Postoperative variables included hospital stay (days), postoperative hypotension, postoperative complications, Clavien–Dindo classification, blood transfusion, mortality, and PASS (Pheochromocytoma of the Adrenal Gland Scaled Score).

#### Definition of outcomes (endpoints) and measurements

The outcomes were the incidence and risk factors for intraoperative HDI in the LRA and LLA procedures. After adrenergic receptor blockade in the pre-induction phase, supine systolic/diastolic blood pressure was < 130/80 mmHg, systolic blood pressure in the upright position was between 90 and 110 mmHg, and heart rate was < 80 beats per min in the supine position and < 100 beats per min while standing [16]. Tumour sizes were recorded according to preoperative CT reports, and tumours ≥ 5 cm were considered large [29]. Intraoperative HDI was defined as SBP > 200 mmHg for more than 1 min or mean arterial pressure (MAP) < 60 mmHg, requiring intravenous vasopressors or vasodilators to maintain normal blood pressure intraoperatively [12]. In this study, we examined the HDI during anaesthesia due to the likelihood of hypertensive episodes during endotracheal intubation [12]. Intraoperative tachycardia (frequency,  > 100 bpm). Maintained Postoperative hypotension was defined as the need for continuous vasopressor infusion to maintain SBP ≥ 90 mmHg [14]. We used the Clavien–Dindo classification to study morbidity [30]. Operative time was defined as the time (in min) between incision and port-site closure or skin closure in conversion to open adrenalectomy. Diabetes Mellitus was defined according to The American Diabetes Association [31]. EBL (Estimated blood loss) was determined as the amount of fluid (in ml) in the aspirator minus the amount of fluid cleaned plus the amount of fluid soaked in gauze in the event of conversion. The American Society of Anesthesiologists (ASA) scale was graded as I–IV [32]. Biologically aggressive behaviour was assessed using the Pheochromocytoma of the Adrenal Gland Scaled Score (PASS), which classified Pheochromocytoma as biologically aggressive behaviour (PASS ≥ 4) from benign tumours (PASS < 4) [33]. Grading and management of liver and splenic injuries were performed according to the American Association for the Surgery of Trauma Organ Injury Scale [34].

#### Perioperative approaches

All patients underwent a standard operating procedure (SOPS) according to the guidelines [35, 36]. All patients with pheochromocytoma commenced preoperative pharmacological preparations with doxazosin or Bunazocin (a selective alpha-blocker) at an initial dose of 2 mg/day for 2 weeks before surgery, which was later adjusted by endocrinologists based on blood pressure levels, reaching a final dose of 32 mg/day [37]. Phenoxybenzamine (a nonselective, irreversible alpha-blocker) was administered for 3 weeks before surgery. The initial dose was 5 mg daily for 1 week, then 10 mg in two doses daily for a second week, and in the last week, it was raised by 10 mg every day as needed to reach the indicated target criteria [38]. Atenolol (Beta-blockers) at an initial dose of 25 mg/day and a final dose of 50 mg/day) was added in cases of tachycardia episodes after at least 3 days of alpha blockade administration to avoid hypertensive crises due to beta-blockade without alpha blockade [37]. The most common perioperative phases associated with HDI include endotracheal intubation, pneumoperitoneum creation, and intraoperative compression of the tumour [39]. Intraoperative hemodynamic monitoring and treatment have been recommended in these guidelines [16, 40, 41]. During adrenalectomy, alpha-blockers (urapidil) and beta-blockers (labetalol) are administered to manage intraoperative hypertension and sinus tachycardia, respectively. Hypotension during and after surgery was treated with volume expansion or norepinephrine infusion if necessary. When the main adrenal vein was clamped, antihypertensive and beta-blocker administration was stopped and hemodynamics were reassessed. Laparoscopic transperitoneal lateral total adrenalectomy was performed as previously described [42]. All surgeries were performed by experienced endocrine surgeons who had performed > 30 laparoscopic adrenalectomies, as previously reported as the minimum number for a completed learning curve of this procedure [43]. Our anaesthetists were cautious during the induction of pneumoperitoneum due to potential hypertension and tachycardia resulting from the massive release of catecholamines [44].

Three trocars were used for LLA and four trocars were used for LRA. The abdominal cavity was insufflated to 14 mmHg (Mercury) using CO_2_ and subsequently reduced to 12 mmHg during the dissection of the pheochromocytoma. The trocars were a supraumbilical 10 mm trocar [camera port], two subcostal ports (10 and 5 mm in the midclavicular and anterior axillary line), and an additional 5 mm trocar placed below the xiphoid for endoretractor liver traction [in the right-sided laparoscopic adrenalectomy]. On the right side, the duodenum was retracted downwards, the right lobe of the liver was retracted upward, and extensive dissection of the hepatodiaphragmatic ligament was performed for complete visualization of the inferior vena cava (IVC) up to the liver hilum. The inferior vena cava was identified and traced to the gland to identify and ligate the right adrenal vein as it entered the inferior vena cava. On the left side, the renal hilum was approached directly to expose the left renal vein, which was then identified and ligated. Dissection stops when there is intraoperative hypertension due to tumour compression or intraoperative hypotension after tumour excision to allow anaesthetists to control the HDI. The adrenal arteries and small adrenal veins were controlled using a harmonic scalpel or LigaSure, a vessel-sealing device, Covidien-Medtronic (clipless adrenalectomy) [45], while a titanium clip or a Hemo-lok clip (Weck Closure Systems, Research Triangle Park, NC, USA) was used to control the large adrenal vein. All specimens were retrieved using an endoscopic pouch (U.S. Surgical, Norwalk, CT, USA) and were sent for histopathological examination. Drainage was selectively used. Postoperatively, all patients were monitored in a high-dependency unit and provided standard postoperative care, including enhanced recovery, early mobilization, and oral ingestion.

### Statistical methods

Statistical analyses were performed using SPSS 28 (IBM Corp., Armonk, New York, USA) and the Kolmogorov–Smirnov test for data visualization and quantitative data analysis for normality. We used the independent *t*-test or Mann–Whitney *U*-test to compare quantitative variables’ normality and non-normality distribution among the groups. Chi-squared or Fisher’s exact tests were used to compare categorical data. ROC analysis was performed for tumor size to predict intraoperative HDI. We also calculated the diagnostic indices and areas under the curve with 95% confidence intervals. Univariate and multivariate logistic regression to predict intraoperative HDI and odds ratios (ORs) with 95% CI). All statistical tests were 2-tailed, and significance was set (*p* < 0.05). The selection of variables in the model was based on clinical experience, which produced a better model.

## Results

### Baseline characteristics

Two hundred seventy-one patients (*n* = 271) were included and divided into LRA (*n* = 122) and LLA (*n* = 149) groups. Table 1 shows baseline patient data. No case of hemodynamic instability occurred during endotracheal intubation or pneumoperitoneum creation. No significant differences were observed between the LRA and LLA groups at the preoperative baseline, except that Body Mass Index (BMI) (32 ± 4 vs. 31 ± 4 kg/m^2^, *p* = 0.047) and 24 h urinary epinephrine (93 ± 24 vs. 85 ± 14 mg/24 h, *p* < 0.001) were higher in the LRA group. The mean ages were 45 ± 9 years and 44 ± 9 years, respectively (*p* = 0.685). Median tumour size was 5.1 cm (2.2–10) vs. 4.7 cm (2.3–9) (*p* = 0.903), and 63 (51.6%) vs. 62 (41.6%) patients showed a tumour diameter ≥ 5 cm (*p* = 0.099). Retrocaval pheochromocytoma was present in 13 (10.7%) patients in the LRA group.

**Table 1:** Baseline preoperative characteristics in the studied groups underwent laparoscopic right adrenalectomy (LRA) and laparoscopic left adrenalectomy (LLA)

	LRA (*n* = 122)	LLA (*n* = 149)	*p* value
Age (years) (mean ± SD)	45 ± 9	44 ± 9	0.685
Sex *n* (%)			
Males	69 (56.6%)	91 (61.1%)	0.452
Females	53 (43.4%)	58 (38.9%)	
Smoking *n* (%)	34 (27.9%)	36 (24.2%)	0.488
Body mass index (mean ± SD)	32 ± 4	31 ± 4	0.047*
Tumor size (cm) median (range)	5.1 (2.2–10)	4.7 (2.3–9)	0.903
Tumor size *n* (%)			
< 5 cm	59 (48.4%)	87 (58.4%)	0.099
≥ 5 cm	63 (51.6%)	62 (41.6%)	
Retrocaval pheochromocytoma *n* (%)	13 (10.7%)	–	–
ASA *n* (%)			0.711
ASA II	72 (59%)	81 (54.4%)	
ASA III	34 (27.9%)	48 (32.2%)	
ASA IV	16 (13.1%)	20 (13.4%)	
Comorbidities *n* (%)			
DM	14 (11%)	17 (11%)	
CHD	9 (7%)	8 (5%)	
HTN	70 (57%)	82 (55%)	0.527
Predominant symptom *n* (%)			
Headache	15 (12.3%)	17 (11.4%)	0.703
Hypertension	65 (53.3%)	75 (50.3%)	
Palpitation	22 (18%)	24 (16.1%)	
Sweating	20 (16.4%)	33 (22.1%)	
Preinduction hemodynamic data mean ± SD			
Preop SBP before alpha blocker	144 ± 7	143 ± 5	0.141
Preop DBP before alpha blocker	93 ± 5	92 ± 5	0.619
Preop SBP after alpha-blocker	122 ± 6	123 ± 5	0.470
Preop DBP after alpha-blocker	74 ± 6	74 ± 6	0.914
Preoperative medical preparation a-Type of alpha-blocker *n* (%)			
Bunazocin	8 (6.6%)	18 (12.1%)	
Doxazocin	59 (48.4%)	52 (34.9%)	0.051
Phenoxybenzamine	55 (45.1%)	79 (53.0%)	
b-Beta-blocker *n* (%)			
Yes	21 (17.2%)	20 (13.4%)	0.386
No	101 (82.8%)	129 (86.6%)	
Antihypertensive drugs number *n* (%)			
One	78 (63.9%)	100 (67.1%)	
Two or more	44 (36.1%)	49 (32.9%)	0.583
24-h urine analysis mean ± SD			
Epinephrine (*n* ≤ 24 mg)	93 ± 24	85 ± 14	< 0.001*
Nor epinephrine (*N* ≤ 66 mg)	129 ± 12	128 ± 11	0.722
Fractionated metanephrine & normetanephrine (*n* = 0–1.2 mg/day)	3.3 ± 0.7	3.2 ± 0.7	0.200
Plasma epinephrine (pg/ml) (*n* ≤ 0.4 ng/ml)	131 ± 21	134 ± 21	0.178
Plasma norepinephrine (pg/ml) (*n* ≤1.6 ng/ml)	758 ± 132	754 ± 127	0.819

*ASA* American society of anesthesiologists, *SBP* systolic blood pressure, *DBP* diastolic blood pressure, *DM* diabetes mellitus, *CHD *coronary heart diseases, *HTN* hyspertension

*Significant *p*-value

### Intraoperative and postoperative differences between left adrenalectomy (LLA) and laparoscopic right adrenalectomy (LRA)

Table 2 shows intraoperative and postoperative data. The LLA approach resulted in shorter operative time (113 ± 21 vs. 131 ± 28 min, *p* < 0.001), less blood loss (131 ± 73 vs. 202 ± 85 ml, *p* < 0.001), lower blood transfusion rates 5 patients (3%) vs. 20 patients (16%), (*p* = 0.001), lower median hospitalization duration 3 (2–5) vs. 4 days (3–6), (*p* < 0.001), and lower early postoperative complications 12 (8.1%) vs. 28 patients (23%), (*p* < 0.001), and lower PASS (Pheochromocytoma of the Adrenal Gland Scaled Score) score ≥ 4 (53(35.6%) vs. 59(48.4%), (*p* = 0.033). The incidence of intraoperative HDI was significantly higher in the LRA group than in the LLA (27% vs. 9.4%, *p* < 0.001). Intraoperative hypertensive crisis was the most common in both groups [19/33 (57.6%) vs. 6/14 (42.9%)]. The median number of episodes of intraoperative HDI was statistically significantly higher in LRA than in LLA [5(3–6) vs. 3(2–6), *p* = 0.01]. There is statistically significant higher intraoperative systolic blood pressure during intraoperative HDI in LRA than in LLA (*p* = 0.001). After Pheochromocytoma resection, intraoperative hypotension occurred in seven patients (21.2%) and 4 patients (28.6%), respectively (*p* = 0.665). Postoperative maintained hypotension was significantly higher in the LRA 14/122 (11.5%) vs. 2/149 (1.3%) (*p* < 0.001). 15 (12.3%) and 9 (6%) patients were converted to laparotomy during LRA and LLA, respectively. Blood transfusion was indicated in cases of intraoperative bleeding during LRA: Intraoperative adrenal vein bleeding (7 patients), IVC bleeding (1 patient), and liver injury (3 patients). Furthermore, blood transfusion was indicated in cases of conversion, as in uncontrollable bleeding from the adrenal veins (5 patients), uncontrolled bleeding from the IVC (1 patient), and in 3 patients by recommendations of the anaesthetists to correct postoperative hypotension. Therefore, 20 patients received blood transfusions.

**Table 2:** Intraoperative and postoperative findings in the studied groups underwent laparoscopic right adrenalectomy (LRA) and laparoscopic left adrenalectomy (LLA)

	LRA (*n* = 122)	LLA (*n* = 149)	*p* value
Operative time (min) mean ± SD	131 ± 28	113 ± 21	< 0.001*
Estimated blood loss (ml) mean ± SD	202 ± 85	131 ± 73	< 0.001*
Blood transfusion *n* (%)	20 (16%)	5 (3%)	0.001*
Intraoperative complications *n* (%)			0.082
Intraoperative acidosis	7 (5.7%)	3 (2)	
Intraoperative adrenal vein bleeding	7 (5.7%)	8 (5.4)	
IVC bleeding	1(0.8%)	0(0%)	
Liver injury	3 (2.5%)	0 (0%)	
Serosal tear of the colon	1 (0.8%)	0 (0%)	
Splenic injury	0 (0%)	1 (0.7%)	
No complications	103 (84.5%)	137 (91.9%)	
Intraoperative hemodynamic instability* n* (%)	33 (27%)	14 (9.4%)	< 0.001*
Intraoperative hemodynamic instability forms *n* (%)			0.665
Hypertensive crisis	19 (57.6%)	6 (42.9%)	
Hypotension	7 (21.2%)	4 (28.6%)	
Tachycardia	7(18.2%)	4(28.6%)	
Number of episodes of intraoperative hemodynamic instability. Median(range)	5(3–6)	3(2–6)	0.01*
Intraoperative systolic blood pressure during hypertensive episodes of intraoperative hemodynamic instability (mmHg) median (range)	263(238–286)	240(235–251)	0.001*
Postoperative maintained hypotension			< 0.001*
Incidence *n* (%)	14 (11.5%)	2 (1.3%)	
Patients received preoperative phenoxybenzamine	7(50%)	0(0%)	
Patients received preoperative doxazocin	7(50%)	2(100%)	
Conversion *n* (%)	15 (12.3%)	9 (6%)	0.071
Causes of conversion *n* (%)			0.209
Recurrent hemodynamic instability during dissection	7 (46.7%)	3 (33.3%)	
Adhesion with difficult dissection	2 (13.3%)	4 (44.4%)	
Uncontrollable bleeding from adrenal veins	5 (33.3%)	1 (11.1%)	
Uncontrollable bleeding from IVC	1 (6.7%)	0 (0%)	
Uncontrollable bleeding from splenic injury	0 (0%)	1 (11.1%)	
Early postoperative complications (30 days)			< 0.001*
Abdominal abscess	1 (0.8)	1 (0.7)	
Acute myocardial infarction	0 (0)	1 (0.7)	
Bleeding	2 (1.6)	2 (1.3)	
Colonic injury	0 (0)	1 (0.7)	
Hyperkalemia	0 (0)	2 (1.3)	
Hypotension	14 (11.5)	2 (1.3)	
Ileus	9 (7.4)	2 (1.3)	
Pneumonia	1 (0.8)	0 (0)	
Wound infection	1 (0.8)	1 (0.7)	
No complications	94 (77)	137 (91.9)	
Clavien–Dindo classification *n* (%)			< 0.001*
Grade 0	94 (77%)	137 (91.9%)	
Grade I	1 (0.8%)	1 (0.7%)	
Grade II	25 (20.5%)	8 (5.4%)	
Grade III	1 (0.8%)	2 (1.3%)	
Grade IV	1 (0.8%)	1 (0.7%)	
Treatment of early complications			0.025*
Conservative + k^+^ losing diuretics	0 (0)	2 (16.7)	
Conservative + antibiotic	1 (3.6)	1 (8.3)	
Conservative + blood transfusion	2 (7.1)	2 (16.7)	
Conservative + cardiac support	0 (0)	1 (8.3)	
Conservative + IV fluid + Ryle	9 (32.1)	2 (16.7)	
Conservative + respiratory support	1 (3.6)	0 (0)	
Postoperative fluid and vasopressor	14 (50)	2 (16.7)	
Radiological drainage	1 (3.6)	1 (8.3)	
Surgical re-intervention	0(0%)	1(8.3%)	
Postoperative hospital stay (days) median (range)	4 (3–6)	3 (2–5)	< 0.001*
Postoperative mortality	2 (1.6%)	0 (0%)	0.202
PASS score *n* (%)			0.033*
< 4	63 (51.6)	96 (64.4)	
≥ 4	59 (48.4)	53 (35.6)	

*IVC* Inferior vena cava, *PASS* pheochromocytoma of the adrenal gland scaled score

*Significant *p*-value

Intraoperative complications did not differ significantly between the groups (*p* = 0.082). The most common intraoperative complications in the LRA group were seven intraoperative acidosis (5.7%) and seven intraoperative adrenal vein bleeding (5.7%), followed by 3 patients with minor liver injuries, which were left untreated or treated with electrocautery, followed by one serosa tear of the colon that was repaired with laparoscopic suturing. Intraoperative complications in the LLA group included 8 patients of intraoperative adrenal vein bleeding, 3 patients of intraoperative acidosis, and 1 patient of splenic injury requiring open conversion and splenectomy. Most postoperative complications were Clavien–Dindo grade II in both groups and were significantly higher in the LRA 25 (20.5%) vs. 8 patients (5.4%) (*p* < 0.001). There were two postoperative deaths (1.6%) in the LRA 10 days after surgery due to acute respiratory failure and myocardial infarction. Reoperation for early complications in LLA occurred in 2 patients who were diagnosed with postoperative colonic injury.

### Regression analysis for the prediction of hemodynamic instability

Univariate and multivariate analyses of the predictors of hemodynamic instability are summarized in Table 3. Univariate analysis was performed for all clinically known risk factors for intraoperative hemodynamic instability, including age, sex, BMI, ASA, tumour size, tumour side, comorbidities, preoperative systolic blood pressure, phenoxybenzamine use, beta-blocker use, intraoperative complications, plasma epinephrine, and plasma norepinephrine. The analysis revealed that BMI (OR = 1.127, 95% CI 1.031–1.232, *p* = 0.008), tumour size (OR = 10.92, 95% CI 5.25–22.715, *p* < 0.001), right-sided tumours (OR = 3.575, 95% CI 1.811–7.058, *p* < 0.001), comorbidities (OR = 29.138, 95% CI 6.759–96.934, *p* < 0.001), and preoperative systolic blood pressure (OR = 1.301, 95% CI 1.191–1.421, *p* < 0.001) were significant predictors of intraoperative HDI. All significant variables at the univariate level were included in a multivariate logistic regression analysis, which revealed that a 1 cm increase in the tumour size was associated with 11 times increased risk of hemodynamic instability (OR = 11.019, 95% CI 3.996–30.38, *p* < 0.001). A right-sided tumour was associated with about 6 times increased risk of hemodynamic instability (5.625; 95% CI 1.147–27.577; *p* = 0.033). Comorbidities were associated with about 8 times increased risk of hemodynamic instability (OR = 7.918, 95% CI 1.323–47.412, *p* = 0.023). One mmHg increase in the preoperative systolic blood pressure was associated with a 26.5% increased risk of hemodynamic instability (OR = 1.265, 95% CI 1.07–1.495, *p* = 0.006).

**Table 3:** Univariate and multivariate logistic regression analysis to predict intraoperative hemodynamic instability

	Univariate		Multivariate	
	OR (95% CI)	*p*-value	OR (95% CI)	*p*-value
Age (years)	1.006 (0.971–1.042)	0.734	–	–
Sex (ref: female gender)	1.276 (0.665–2.448)	0.463	–	–
BMI	1.127 (1.031–1.232)	0.008**	1.125 (0.887–1.427)	0.331
ASA (ref: ASA II)			–	–
ASA III	1.303 (0.648–2.620)	0.458	–	–
ASA IV	1.297 (0.510–3.299)	0.585	–	–
Tumor size (cm)	10.92 (5.25–22.715)	< 0.001*	11.019 (3.996–30.38)	< 0.001*
Right side tumor	3.575 (1.811–7.058)	< 0.001*	5.625 (1.147–27.577)	0.033*
Comorbidities [DM, hypertension, CHD]	29.138 (8.759–96.934)	< 0.001*	7.918 (1.323–47.412)	0.023*
Preoperative SBP	1.301 (1.191–1.421)	< 0.001*	1.265 (1.07–1.495)	0.006*
Phenoxybenzamine use	0.793 (0.422–1.493)	0.473	–	–
Beta-blockers use	1.683 (0.76–3.726)	0.199	–	–
Intraoperative complications	0.497 (0.144–1.714)	0.269	–	–
Plasma epinephrine (pg/ml)	1.003 (0.988–1.018)	0.706	–	–
Plasma norepinephrine (pg/ml)	0.999 (0.997–1.002)	0.462	–	–ss

*OR* Odds ratio, 95% *CI* 95% confidence interval, *BMI* body mass index, *ASA* American society of anaesthesiology, *SBP* systolic blood pressure, *SBP* systolic blood pressure, *CHD* coronary heart disease, *DM* diabetes mellitus, *VMA* valenyle mandelic acid

*Significant *p*-value

### Size cutoff for intraoperative hemodynamic instability between left adrenalectomy (LLA) and laparoscopic right adrenalectomy (LRA)

**Fig. 2:**
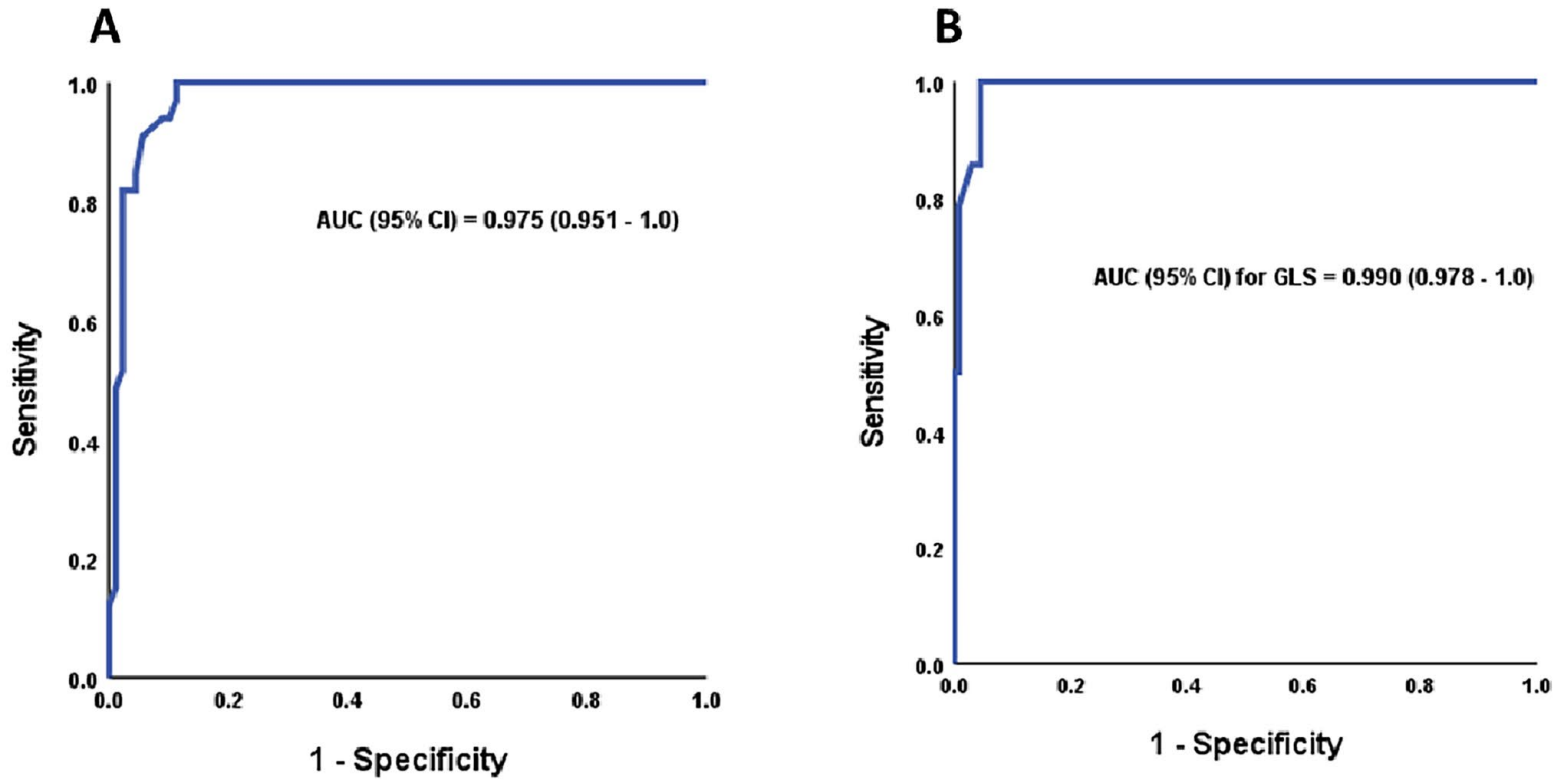
ROC analysis for tumour size to predict intraoperative hemodynamic instability in **A** right adrenalectomy and **B** left adrenalectomy

We performed a receiver operating characteristic (ROC) analysis of tumour size to predict intraoperative hemodynamic instability. In LRA, the best cutoff was  ≥ 5.65 cm, at which the sensitivity and specificity were 100 and 88.8%, respectively (Fig. 2a), while in LLA, the best cutoff was ≥ 6.55 cm, at which the sensitivity and specificity were 100 and 95.6%, respectively (Fig. 2b).

### Analysis subgroup of tumour size

Supplementary Table 1 shows baseline, intraoperative and postoperative findings according to tumour size.

## Discussion

This study compared the incidence and risk factors of intraoperative HDI between laparoscopic left adrenalectomy (LLA) and laparoscopic right adrenalectomy (LRA) in a large cohort of patients with benign pheochromocytomas. Few studies have compared adrenalectomy on both sides; however, they did not compare the differences in the incidence and risk factors of intraoperative HDI between both approaches in a large cohort [15, 17, 18, 20, 21]. In our study, HDI was significantly higher in the LRA than in the LLA (27 vs. 9.4%). RT-sided PHEOS is a risk factor for intraoperative HDI.

Hemodynamic instability is one of the most frequent adverse events observed in patients undergoing laparoscopic pheochromocytoma removal. The incidence of intraoperative HDI was 27% in LRA and 9.4% in LLA in our series, which was lower than reported previously by Pisarska (43.8%) [15]. Pisarska et al. did not specify which side had a higher perioperative HDI. Compared with the study by Pisarska, the lower incidence of intraoperative HDI in our study may be due to variations in sample size, patient selection, exclusion of cases with previous abdominal surgery, improved preoperative medical preparation [16], anaesthesia strategy, and meticulous surgical techniques [12]. Furthermore, our initial surgical aim was to perform initial control of the adrenal vein, aiming at the systematic devascularization of pheochromocytoma with minimal manipulation of the adrenal gland. The incidence of intraoperative HDI in our study was significantly higher in LRA than in LLA due to the retrocaval position of the tumour, a short right adrenal vein that drains into the IVC in the posterolateral position, larger tumour size, and the top pole of the adrenal gland remaining buried beneath the caudate lobe of the liver in obese or “large” liver patients. Owing to the retrocaval position of the right adrenal gland, during LRA, we initially placed an aspirator between the adrenal gland and vena cava to carefully retract the tumour and avoid inferior vena cava injury at the expense of neoplasm compression. Additionally, owing to its shorter length, direct drainage into the inferior vena cava, and location under the liver bed, the right adrenal vein can be more challenging to expose and easy injury with difficulty to control during LRA, which leads to tumour compression during attempts to control bleeding [46]. Logistic regression analysis in this study confirmed our opinion that right-sided Pheochromocytoma is a risk factor for intraoperative HDI, in contrast to a study that found an equivalent risk (*p* = 0.4667) [15]. On the other hand, other studies stated that the left side could also be difficult because of its proximity to the tail of the pancreas, splenic vasculature, unforgiving nature of the spleen itself and the surgical point of dissection of the left renal hilum for ligation of the left adrenal vein; therefore, they found that HI is more common in left-sided tumours [18, 19]. During LLA, we mobilized the splenic flexure of the colon, and the downward movement of the spleen by gravity by patient tilt helped us significantly expose the left adrenal area with minimal manipulation of the pheochromocytoma. The most common cause of conversion during LLA was extensive adhesion at the left adrenal region with difficult identification of the proper anatomy, and conversion was for patient safety without much pheochromocytoma compression. Unstandardized perioperative anaesthesia, surgical care, and varied HDI definitions may explain this variability as additional factors of differences in HDI in both the LRA and LLA.

Exploring the risk predictors of intraoperative HDI may improve the treatment outcomes. However, the role of pheochromocytoma size as a common predictor of HDI remains controversial. Currently, the maximum tumour diameter of the LA is 22.5 cm [47]. Our institutions used transperitoneal laparoscopic adrenalectomy in all scheduled adrenalectomies, regardless of tumour size, and found that tumour size is a risk factor for intraoperative HDI; this result is similar to that of other studies [20, 21] in contrast to another study that reported that tumour size was not a predictor of intraoperative HDI [22]. Surprisingly, one study stated that small-sized tumours are associated with HDI more than larger-sized tumours and are attributed to the necrosis of tumour occurrence as it enlarges and subsequently becomes less functioning [48]. However, these previous studies did not address the side effects of HDI. As the gland size increased, the length of the main vein shortened, the field of vision became limited, and the risk of increased vascularity and adhesion to surrounding structures increased. Manipulation of the neoplasm and delayed ligation of the adrenal vein would lead to squeezing of the gland and release of catecholamines into the circulation. We had difficulty accessing the adrenal vein underneath the larger tumours during LRA, which made early ligation more challenging. Hemodynamic instability during LRA started at a smaller tumour size than that during LLA, as evidenced by the ROC curve, which revealed that the cutoff for neoplasm size for HDI in LRA was smaller than that for LLA (≥ 5.65 vs ≥ 6.55 cm, respectively).

Some disorders compromise cardiovascular function, thus affecting intraoperative hemodynamic parameters. Bai et al. [49] demonstrated that BMI and coronary heart disease (CHD) were predictors of HDI; however, there was no significant difference between LRA and LLA (*p* = 0.333). Our multivariate analysis showed a causal connection between comorbidities (Diabetes Mellitus and CHD) and HDI in LRA and LLA. Higher arterial stiffness in diabetes mellitus affects cardiovascular system function and structure [50]. Furthermore, hypertensive patients have higher arterial stiffness than normotensive patients do, making them more susceptible to episodes of hypertension. Alpha-blockers control blood pressure to prevent intraoperative HDI. Thompson et al. showed that preoperative alpha-blockers and beta-blockers were not risk factors for intraoperative HDI [51]. Our univariate regression showed that phenoxybenzamine use before surgery reduced intraoperative HDI in the LRA and LLA by 30%, but the multivariate analysis did not. The study may have had few intraoperative HDI to determine the alpha-blocker relationship.

This study has several limitations. First, this retrospective study may have had selection bias. Second, our results may not apply to open adrenalectomies because the current study only examined patients who underwent LA. Third, we did not have data on the durations of the intraoperative HDI. Additionally, we have a limitation in that the number of patients experiencing hemodynamic instability was not large. Therefore, the model was clinically built, focusing away from statistical significance and including only clinically known factors that might affect the outcome. The best model is built on a clinical basis, not just statistical significance without any clinical sense. Despite these limitations, up to our knowledge, this study is the first multi-institution comparative cohort study to compare the incidence and risk factors of intraoperative HDI between LRA and LLA (side-specific factors for intraoperative hemodynamic instability) in a large cohort.

## Conclusion

Compared to LLA, LRA is associated with a higher perioperative HDI rate. Pheochromocytoma size, preoperative comorbidities, high preoperative systolic blood pressure, and right-sided pheochromocytoma were associated with higher HDI during pheochromocytoma resection. Preoperative evaluation of these risk factors is of great value for surgeons and anaesthetists for evaluating the risk of HDI, making a surgical plan, reducing the HDI rate, and improving the intraoperative situation and postoperative outcomes. Pheochromocytoma size > 5.6 cm could be performed safely, but surgeons and anaesthetists should be cautious of the higher risk of HDI with all precautions taken. We recommend that to reduce the incidence of HDI during LRA for pheochromocytoma, the pressure on the pneumoperitoneum should be reduced when operating on the right side, greater mobilization of the liver should be performed initially to expand the surgical field, and laparoscopic adrenalectomy for pheochromocytoma should be performed in high-flow centres with experienced endocrine surgeons and anaesthetists.

### Supplementary Information

Below is the link to the electronic supplementary material.

Supplementary file1 (DOCX 27 kb)

## Data Availability

Availability of data and material are available on request from the corresponding author.

## Declarations

### Research Involving Human and Animal Participants

All procedures were carried out in compliance with the 1964 Helsinki Declaration.
