# Supplementary material for: Side-specific factors for intraoperative hemodynamic instability in laparoscopic adrenalectomy for pheochromocytoma: a comparative study
Source: Surg Endosc. 2024 Jul 1;38(8):4571–82. doi: 10.1007/s00464-024-10974-w (PMC11289338; doi:10.1007/s00464-024-10974-w)
Supplement: Supplementary file 1 — Supplementary file1 (DOCX 27 kb) [file 464_2024_10974_MOESM1_ESM.docx]

**Supplementary Table 1: Baseline, intraoperative, and postoperative findings according to tumor size**

|  |  | **Tumor size** | |  |
| --- | --- | --- | --- | --- |
|  |  | **<5 cm** | **≥ 5 cm** | **P-value** |
| **Age (years)** | Mean ±SD | 45 ±9 | 44 ±9 | 0.723 |
| **Side** |  |  |  |  |
| Right | n (%) | 59 (40.4) | 63 (50.4) | 0.099 |
| Left | n (%) | 87 (59.6) | 62 (49.6) |  |
| **Sex** |  |  |  |  |
| Males | n (%) | 80 (54.8) | 80 (64) | 0.124 |
| Females | n (%) | 66 (45.2) | 45 (36) |  |
| **Smoking** | n (%) | 38 (26) | 32 (25.6) | 0.936 |
| **BMI** | Mean ±SD | 32 ±4 | 32 ±4 | 0.775 |
| **Retrocaval pheochromocytoma** | n (%) | 0 (0) | 13 (20.6) | **<0.001** |
| **ASA** |  |  |  |  |
| ASA II | n (%) | 86 (58.9) | 67 (53.6) | 0.593 |
| ASA III | n (%) | 43 (29.5) | 39 (31.2) |  |
| ASA IV | n (%) | 17 (11.6) | 19 (15.2) |  |
| **Comorbidities** | n (%) | 46 (31.5) | 73 (58.4) | **<0.001** |
| **Preoperative predominant clinical symptoms** |  |  |  |  |
| Headache | n (%) | 19 (13) | 13 (10.4) | 0.186 |
| HTN | n (%) | 67 (45.9) | 73 (58.4) |  |
| Palpitation | n (%) | 26 (17.8) | 20 (16) |  |
| Sweating | n (%) | 34 (23.3) | 19 (15.2) |  |
| **Preoperative SBP (before alpha blocker)** | Mean ±SD | 142 ±6 | 145 ±6 | **<0.001** |
| **Preoperative DBP (before alpha blocker)** | Mean ±SD | 92 ±5 | 93 ±5 | 0.648 |
| **Preoperative SBP (after alpha blocker)** | Mean ±SD | 122 ±5 | 122 ±6 | 0.904 |
| **preoperative DBP (after alpha blocker)** | Mean ±SD | 74 ±6 | 74 ±6 | 0.646 |
| **Alpha blocker** |  |  |  |  |
| Bunazocin | n (%) | 16 (11) | 10 (8) | 0.693 |
| Doxazocin | n (%) | 58 (39.7) | 53 (42.4) |  |
| Phenoxypenzamine | n (%) | 72 (49.3) | 62 (49.6) |  |
| **Beta blocker** |  |  |  |  |
| yes | n (%) | 17 (11.6) | 24 (19.2) | 0.084 |
| No | n (%) | 129 (88.4) | 101 (80.8) |  |
| **Number of antihypertensive drugs** |  |  |  |  |
| One | n (%) | 111 (76) | 67 (53.6) | **<0.001** |
| Two or more | n (%) | 35 (24) | 58 (46.4) |  |
| **24 hr urinary epinephrine (microgram/24)** | Mean ±SD | 82 ±12 | 96 ±23 | **<0.001** |
| **24 hr urinary nor epinephrine(microgram/24)** | Mean ±SD | 129 ±11 | 128 ±12 | 0.755 |
| **24 hr urinary fractionated metanephrine and nor metanephrine(mg/24)** | Mean ±SD | 3.2 ±0.7 | 3.2 ±0.7 | 0.955 |
| **plasma epinephrine(pg/ml)** | Mean ±SD | 134 ±21 | 132 ±21 | 0.501 |
| **plasma nor epinephrine(pg/ml)** | Mean ±SD | 759 ±129 | 752 ±129 | 0.636 |
| **Operative time(min)** | Mean ±SD | 137 ±20 | 116 ±32 | **<0.001** |
| **Blood loss (ml)** | Mean ±SD | 157 ±79 | 117 ±94 | 0.54 |
| **Intraoperative hemodynamic instability** | n (%) | 0 (0) | 47 (37.6) | **<0.001** |
| **Intraoperative complications** |  |  |  |  |
| Intraoperative acidosis | n (%) | 6 (4.1) | 4 (3.2) | 0.441 |
| Intraoperative bleeding | n (%) | 11 (7.5) | 4 (3.2) |  |
| Liver injury | n (%) | 2 (1.4) | 1 (0.8) |  |
| Serosal tear of the colon | n (%) | 0 (0) | 1 (0.8) |  |
| Splenic injury | n (%) | 1 (0.7) | 0 (0) |  |
| No | n (%) | 126 (86.3) | 115 (92) |  |
| **Conversion** | n (%) | 9 (6.2) | 15 (12) | 0.092 |
| **Hospital stay** | Median (range) | 4 (2 - 5) | 4 (2 - 6) | 0.680 |
| **Mortality** | n (%) | 2 (1.4) | 0 (0) | 0.501 |
| **PASS score** |  |  |  |  |
| <4 | n (%) | 99 (67.8) | 60 (48) | **<0.001** |
| >=4 | n (%) | 47 (32.2) | 65 (52) |  |
